# A qualitative analysis of the barriers and facilitators of HIV counselling and testing perceived by adolescents in South Africa

**DOI:** 10.1186/s12913-015-0922-0

**Published:** 2015-06-30

**Authors:** Michael Strauss, Bruce Rhodes, Gavin George

**Affiliations:** Health Economics and HIV and AIDS Research Division (HEARD), University of KwaZulu-Natal, 4th Floor, J-Block, University Drive, Westville, 4000 South Africa; School of Accounting, Economics and Finance, University of KwaZulu-Natal, 3rd Floor, J-Block, University Drive, Westville, 4000 South Africa

**Keywords:** HIV, Adolescents, HIV counselling and testing, Demand creation

## Abstract

**Background:**

Youth in South Africa have been identified as a high-risk group for contracting HIV. In response, the South African Integrated School Health Policy (ISHP) has been developed with the aim of guiding the provision of comprehensive healthcare services within South African schools. Accordingly, the scale-up of HIV counselling and testing (HCT) in high schools is a priority. This study examines the factors affecting the utilisation of HCT services amongst learners in high schools in the KwaZulu-Natal province of South Africa.

**Methods:**

Focus group discussions were conducted in 12 rural schools in the Vulindlela sub-district of uMgungundlovu in KwaZulu-Natal. A total of 158 randomly selected learners took part, aged 16 years and older from grades 10, 11 and 12. Qualitative analysis was conducted using the framework approach, providing a systematic structure allowing for a priori and emergent codes, with social cognitive theory as a theoretical framework.

**Results:**

The stigma and discrimination attached to testing, along with the inherent fear of a positive result were the biggest barriers to HCT uptake. Fear and the subsequent negative beliefs around HCT were borne out of insufficient knowledge. These fears were exacerbated by the perceived or real attitudes of peers, partners and family towards HIV. The prospect of a positive result and the possible resultant societal backlash hinders high and regular uptake of HCT. Stigma and discrimination remain the foremost barriers to HIV testing despite the presence of localised and convenient testing. Interventions aimed at addressing these challenges could increase the demand for HIV testing amongst adolescents.

**Conclusions:**

Increasing education about the importance of HCT and creating awareness about available HCT services will not be enough to increase uptake in schools in South Africa. Efforts to decrease stigma around HIV and HCT by integrating testing into general and sexual reproductive health services offered to youth, and normalising the epidemic within the community could go some way to allaying the fears shrouding testing, if such services are designed with the specific needs of youth in mind. This paper adds to the body of literature informing the design of policy in South Africa aimed at integrating HCT into school health services.

## Background

Youth (15–24 age group^1^) in South Africa have been identified as a high-risk group with HIV prevalence rates estimated at 7,1 % [1]. The KwaZulu-Natal (KZN) province has the second largest population (10,8 million people) and the biggest burden of HIV in the country, both in terms of prevalence (24.7 %) and incidence rates (2.3 %) [2]. Prevalence among youth in KZN is also substantially higher than the national average, estimated at over 15 %, and higher than any other province [3]. Among all communities, the Vulindlela sub-district in Umgungundlovu, KZN, has one of the highest HIV prevalence rates (45.7 %) in South Africa, with a population of approximately 90,000 [4], and is therefore a critical district in terms of HCT service delivery. The provision of HCT targeting school-going youth is therefore a key strategy in managing HIV in this high-prevalence area. Youth-friendly provision of HCT is crucial in ensuring the uptake of HCT services amongst this targeted constituency. The results of this qualitative study examine the barriers to and facilitators of HCT, adding to the understanding of what constitutes youth-friendly HCT services, and the results highlight the important opportunities and threats of including HIV testing in the newly developed South African integrated school health policy (ISHP).

HIV counselling and testing (HCT) is one of the most effective and important interventions for managing the HIV epidemic, and one of the pillars of the South African Investment Case [5]. There is substantial evidence showing the link between increased HCT and reduced HIV incidence [6, 7]. The first objective outlined in the South African National Strategic Plan on HIV, STIs and TB 2012–2016 calls for everyone in the country to voluntarily test for HIV at least once a year [8]. Not only is annual voluntary HIV screening known to offer substantial clinical benefits, it is estimated to be cost-effective because of the positive effects on incidence, even with limited linkage to care and treatment [9].

HCT is available to high school learners through local clinics, hospitals and various mobile testing facilities. Integrating HCT into a healthcare regime for children at an early age needs to be a priority for policy makers, especially as the legal age for consensual sex in South Africa was recently lowered to 12 years of age [10]. South Africa is a global leader in children’s rights legislation; especially regulations relating to the provision of health services to minors, and laws regarding sex and sexual health have changed substantially since the turn of the century. The age of majority (when an individual no longer needs parental consent for any decision) was changed from 21 to 18 years [11]. Any person younger than 18 years may not marry without the written consent of their parents, and consent must also be obtained from the Minister of Home Affairs for boys younger than 18 and girls younger than 15 [12]. The Children’s Act 38 of 2005, as amended by the Children’s Amendment Act 41 of 2007, makes provision for children older than 12 years to access medical surgery and treatment, including contraceptives and HIV testing, without parental consent, and the result of the test along with other medical information may not be divulged to parents in terms of the Act [11].

The legal age of consensual sex, however, remained at 16 years, and plans to begin a scale-up of HCT in schools around South Africa were met with much controversy and debate, in spite of legislation and evidence that supported the roll out of HCT in schools [13]. Following delays and much consultation between stakeholders, a statement was released by the Minister of Health, Aaron Motsoaledi, in October 2012, saying it would be irresponsible to deny pupils access to condoms and HIV testing at schools, and that government would soon launch an Integrated School Health Programme (ISHP) [14–16]. This was followed in early 2013 by the change in legislation legalising consensual sex between children who are between the ages of 12 and 16 [10].

There must be a robust debate around the integration of health services versus stand-alone interventions [17]. However, whilst this debate is important, the focus of delivering healthcare services to school-going adolescents by the public sector in South Africa is moving towards integration through the ISHP [18]. The ISHP includes HCT in the range of services offered to learners, with a focus on offering youth-friendly services. This policy will be instrumental in ensuring youth have access to HIV prevention and treatment services and that these services are offered in a way that will protect as well as educate [18]. Effective execution of the ISHP must ensure that the barriers to HCT uptake amongst youth are mitigated, if it is to achieve its intended purpose.

### Social cognitive theory

**Fig. 1:**
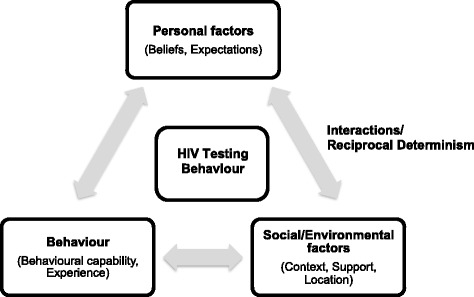
Social cognitive framework

This study examines both the barriers and facilitators affecting the utilisation of HCT services amongst school-going youth using Social Cognitive Theory (SCT) as the theoretical framework [19]. This framework has previously been used in the examination of HIV prevention programmes [20, 21]. The SCT framework identifies three main sources of influence on people’s attitudes, perceptions and intentions to act and their ability to carry out their intentions. These are: individual determinants; behaviour; and environmental or social factors [19]. SCT suggests that ‘behaviours, environmental influences, and beliefs are highly interactive and dependent’ [22]. It is these factors, and particularly the reciprocal determinism that exists between them, which can positively or negatively impact the utilisation of HCT services. An understanding of these influences could therefore inform the design and implementation of HCT services advocated by the ISHP. Figure 1 is a simple representation of social cognitive theory adapted from Bandura’s contribution to SCT in the context of HIV [19].

While SCT has rational cognitive decision-making at the centre of understanding behaviour, it also provides a framework for consideration of the broader context, including the social, cultural and historical constructs in which individuals find themselves making choices, and is wholly relevant to the uptake of HCT. Within the SCT context certain barriers to and facilitators of HCT can be explained.

### Barriers to and facilitators of HIV counselling and testing

Existing literature highlights some of the barriers and facilitators that exist because of the interaction between personal, contextual and behavioural factors. At the personal level, a key finding is the importance of knowledge about testing and HIV. A lack of knowledge can be a significant barrier to testing [23–25]. Young people’s behaviour can affect their beliefs and intentions to test. Individuals perceiving themselves to be at high risk due to unsafe sexual practices and suspecting a positive result were less likely to test [26]. Further studies showed that learners not engaging in sexual activity and therefore not perceiving themselves not to be at risk were also less likely to undergo testing [24, 27]. Young people with a history of regular HCT [28], and those that reported visiting a health clinic more frequently, were found to be more likely to continue to undergo testing [29].

The interaction between personal factors and the context in which learners find themselves influences willingness to undergo HCT. Fear of HIV-related stigma and discrimination, regardless of their HCT result, can be a significant barrier to testing [25, 28]. Support from the community, especially family and friends, however, was found to be an important facilitator of HCT, with research showing that the ability to speak to parents about HIV and AIDS was a significant predictor of willingness to undergo testing [25, 29]. Some programmes have successfully used peer recruiters to increase acceptability and demand for HCT [30]. Concerns about confidentiality are often linked to a fear of stigmatisation [24, 25, 31, 32]. Studies revealed direct trust issues relating to healthcare workers administering the counselling and testing [25], [31]. Delivery of health services is particularly challenging for high school learners, as travel time, long queues and the limited opening hours of facilities often mean learners have to miss classes to access services. For high school learners, especially in rural areas, money can be an important consideration. Offering HCT free of charge has been found to be a significant facilitator for young people, and reducing travel costs by offering testing at mobile HCT stations has also shown to increase demand [33].

This study adds to the currently limited, but growing body of literature that unpacks the complex interrelationship between the underlying factors that influence young people’s willingness to test, while providing insights which would aid in the development and implementation of policy targeting youth in South Africa.

## Methods

### Study setting

This study was conducted between September and November 2012, and June and July 2013, as part of an on-going prospective cohort study to assess behavioural disinhibition following medical circumcision amongst school-going youth in the uMgungundlovu district in KZN. A voluntary medical male circumcision (VMMC) programme beginning in March 2011, run by the Centre for the Aids Programme of Research in South Africa (CAPRISA), had circumcised 2971 boys between January 2010 and July 2012, targeting boys from 42 high schools in the Vulindlela sub-district of uMgungundlovu [30]. Previously, CAPRISA had also run an HCT programme among girls in high schools in the area, which showed the need to increase the provision of school-based HIV testing programmes, as a gateway to treatment and prevention services [34].

HCT was a prerequisite for circumcision in the more recent VMMC programme, with testing offered free of charge to all learners (both males and females) in the schools, whether participating in the study or not. HCT facilities were made available both at the schools through mobile testing facilities and at the CAPRISA clinic in Vulindlela. These facilities were standardised across all the schools included in the study, although learners may also have had experience of testing at other local clinics, hospitals or other service providers.

The qualitative work we present here assessed the success of the HCT programmes, the impact, threats and opportunities that exist in scaling up the provision of sexual healthcare among high school learners in rural areas, as well as the importance of services integrated into school health programmes.

### Sampling framework

Twelve out of the 42 high schools in Vulindlela where the male circumcision programme was run, were selected to participate in this study. Selected schools were peri-urban, e.g. Mlungisi and Insika located 30–45 km from the central business district (CBD) of Pietermaritzburg, and rural, e.g., KwaMncane and Gobindlovu located at least 50 km from the CBD. The schools included in the study were chosen as a representative sample of all the schools in the area, with input in the decision from fieldworkers who lived in Vulindlela and colleagues at CAPRISA. All 12 high schools were under resourced, in need of repair, and servicing learners from very similar socio-economic backgrounds. Public schools in South Africa are categorised into five quintiles based on the availability of resources, and schools in this sample all fall into the lowest three quintiles—all declared to be “no fee schools” and financed by government funding. The Vulindlela community has high levels of poverty, resulting from the limited number of job opportunities and lack of infrastructure.

### Data collection and analysis

Data were collected using focus group discussions (FGDs) in the local Zulu language. FGDs were used, rather than personal interviews, as it was felt that the latter would be too intimidating and not directly comparable to the extant literature. A total of 21 focus groups were conducted, 8 consisting only of randomly selected boys and 8 consisting only of randomly selected girls to ensure learners were comfortable enough to share their views. Each focus group had approximately 6–8 participants aged 16 years and older from grades 10, 11 and 12. There was no upper age limit for this study, as long as participants were still in school, however, school records and results from the broader study indicated an age range for learners in these grades of 16–19 (although one learner self-reported an age of 24 years). An additional five FGDs were held in 2013 to augment the data, with only boys who were attending a circumcision camp, where they were able to undergo HCT and VMMC in a supportive environment.^2^ The HCT service delivery mechanism at the camp was similar to that of the mobile testing facilities offered at schools. A total of 158 learners took part in these focus groups, with a mix of those who had been tested previously and those who had not. The reported data consist largely of quotes that identify themes of response. Referencing each quote by school is not reported, to honour the declaration of anonymity, and identifying the schools would add no value to the results and themes developed as they are not location dependent within this geographical scope, given the insignificant variation of socio-economic background.

The FGD questions were structured into a pre-existing framework aligned with the research objectives. The overarching themes of questions included: (1) barriers to and facilitators of HCT, (2) perceptions about the HCT services available to learners, and (3) the experience of those learners who had attended HCT clinics or mobile counselling and testing facilities. Interviewers facilitated discussion among learners, and specific questions and probes were focused on identifying factors influencing HIV-testing behaviour at the personal and social/environmental levels, as well as on identifying behavioural factors that affect willingness to undergo HCT within the three overarching themes.

FGDs were conducted by three male fieldworkers fluent in both Zulu and English and trained in qualitative interviewing practices. The fieldworkers had previous experience in undertaking research in this community. Whilst the discussions were driven by the questions and themes discussed above, fieldworkers were encouraged to allow the conversation to be guided by the participants.

The FGDs were conducted in Zulu, audio-recorded and transcribed verbatim. Transcripts were then translated into English and coded using NVivo 10 (QSR International, Australia). The framework approach [35], which provides a systematic structure, allowing for a priori and emergent codes, was used as a method of qualitative analysis.

As recommended [36], the transcripts were read twice by the researchers to familiarise themselves with the data before coding began. This was also done to gain a better overall perspective of the context as well as the perceptions and experiences of participants in order to reduce the influence of researchers’ own subjective opinions during analysis of the data. This method of open coding was used to avoid biases in the development of codes based on the researchers’ prior knowledge, beliefs and perceptions about HCT. Opler’s terminology of ‘expressions’ and ‘themes’ was used for coding data [37].

Descriptive codes were used to identify basic expressions found in FGD transcripts and categorised into themes and sub-themes and given more analytical coding labels. Ryan and Bernard’s techniques for identifying themes from basic expressions in the text were used [37].

### Ethics

Ethical clearance for this study was obtained from the Social Sciences Ethics Committee at the University of KwaZulu-Natal (HSS/0649012 M). A waiver of parental consent for learners older than 16 years was granted, given that learners had access to HCT without parental consent in this age group, and given that a waiver of parental consent for learners older than 16 years was granted by the Biomedical Research Ethics Committee for the broader work being undertaken by CAPRISA in the same schools. Written informed consent was obtained from all participants in line with guidelines from the ethics committee and all standard procedures of volunteer participation and confidentiality were followed.

## Results

SCT suggests that the interrelationship and interaction between individuals’ behaviour, their beliefs and the environment in which the individual is situated can influence their health seeking behaviour [19]. Identifying these levers provides insight into how programmes can positively influence health-seeking practices, and specifically, increase the uptake of HCT. This study identifies and categorises these factors, which influence testing behaviour amongst this cohort. Barriers to testing are borne out of individual behaviour and beliefs, whilst environmental factors can both positively and negatively influence testing uptake. Through both inductive and deductive analysis the resistance to testing is revealed, and the reasons underpinning this resistance is explained through the SCT lens.

Fear, or the lack of it, was the dominant theme emerging from the data. This fear of testing or the confidence to test is realised through the interaction between the self and the environment, with behaviour relating to sexual risk, and experience of HCT having an impact on personal beliefs and the decision to test and repeat test. Risky behaviour can influence decisions, and learners who perceive themselves to be at risk are less likely to test, fearing a positive result.

Boy: Those scholars who are involved in night clubs and are promiscuous maybe with people who are said to be infected. Their whole lifestyle might cause that individual not to go for an HIV test, fearing that he is HIV positive he will take his own life.

Girl: Some might be too intimidated to test because they know they have a number of girlfriends, and they are aware that once they have tested positive they would not be as happy as they are currently, they will be constantly thinking of their results.

The quotes above point to the inability of individuals to manage the consequences of a positive HIV result. The lack of knowledge and a perceived lack of support results in these learners avoiding HCT and the likely outcome.

Inadequate knowledge of the epidemic was a barrier to testing. Although comprehensive counselling and education about HIV is offered as part of the HCT package, learners who decline HCT do not access this information.

Girl: I was scared because I didn’t know what we are actually testing for, and if I were positive what would I have done about it; because if I did I would have committed suicide. People say if you are HIV positive, it’s better to die instead of living with the virus because HIV/AIDS is not curable.

The resultant beliefs of learners around a positive result lead to feelings of fear and hopelessness.

Girl: I don’t think it’s best for someone who’s scared to get tested, because you can test and hear that you have HIV/AIDS and then decide to kill yourself. Lots of people kill themselves after hearing about this, but I think it’s good to know your status.

The level of support offered to leaners before and after testing is crucial for the success of increasing the uptake of voluntary testing. Support is not solely sought from the professional healthcare sector, but also from family and friends, as well as acceptance by the wider community. In order to stimulate testing behaviour an environment conducive to seeking such information must exist. A lack of knowledge and perceived lack of support from peers, family, teachers and the community at large were found to be very influential in the decision to test or not.

Although there are concerns about stigma and discrimination, many learners find strength from the support of those who are close and whom they can trust. Results reveal the influence of friends and teachers in increasing HCT uptake.

Boy: I think I have gained the strength from other guys who were going to the tents to test, and I thought that it is not good to be left behind because the guys were willing to go there. Yes it helps if you do things as a group than do it alone.

Boy: We would just go with my mates, and they [the teachers] had a very good way of motivating us by teaching about STI’s and also showing picture of what an infected person looks like.

Whilst results show that teachers can facilitate uptake by encouraging learners to get tested, the role of “peer educators” was perceived to be less effective for some learners.

Girl: Unfortunately, these peer educators do not do anything in motivating us, they really are not effective, on the teachers side, they do try to educate and give us some awareness on it and how should we best behave as youngsters, even to those who are party animals, they also encourage them to use protection in case he is drunk and intend to have sex.

Boy: When you are told on HIV related issues by a peer educator, you tend to say to yourself “how can you tell me about something you have never experienced, the virus that he is talking about? He has no clue on what it does to the body because he has never been sick!”

Family were also found in some cases to be a source of support. While some learners felt it was important that their family did not find out that they had tested, many said their family were supportive and encouraged them to undergo HCT. Some participants even expressed an interest in having the HIV test done at home, indicating the presence of a supportive and non-judgemental environment.

Boy: I think that will be great if the whole family test. It will be good if the test were to be done at our home, because it is rare that families have serious fights disclosing each other’s HIV positive statuses, family might have misunderstandings and fights but I doubt that they can use their statuses against one another.

In order to counter some of the fears associated with HCT, learners stressed the need for appropriate counselling. High-quality counselling addresses a number of different issues, including preparing individuals for testing, and is important in helping learners make responsible health and life decisions. Knowing that counselling is available was found to be an important facilitator influencing a participant’s decision to test.

Boy: You might have a problem when you learn that you are HIV positive, because you were not counselled before your test. I think it’s better to be counselled first, so that you know how to take care of yourself if the results came back positive. It’s better to be counselled first if ever you find that you have this illness. That will avoid you doing something wrong after learning about your result.

Boy: It was useful because I had fear before counselling, because testing was something I never did before, but counselling helped me decide to test.

Leaners who had undergone HCT attested to the support and knowledge they received from the counsellors. They understood that being HIV positive is something that can be managed.

Girl: At the testing centre, you will find counsellors which helps you if you test positive by giving you advice and guidance on how to live positive lifestyle, and they will tell you that if you are HIV positive it doesn’t mean that it’s the end of your life, because there are ARV’s that you can use and live a normal life like everyone. If you know your HIV status, you will be able to know the steps you need to take and if you test negative, there are also guidelines which you will have to take in order to prevent being HIV positive.

Boy: Some are happy about testing, so that they know their status. And if you are positive, it will be easy to follow the route of living with HIV. And if the results are negative, you can be able to avoid yourself from getting HIV.

The experiences of learners at testing centres are a crucial mechanism in ensuring positive feedback to peers. These experiences mitigate against negative beliefs around testing and a positive result. The testing environment is therefore crucial in facilitating broader access to testing from the broader community. Stigma and discrimination, whether real or perceived, remain stumbling blocks to increased testing. Staff at testing facilities should therefore be cognisant of the inherent fears of the communities and constituents they seek to serve.

The length of queuing time was shown to influence the willingness to test where longer queuing times seemed to provoke anxieties.

Boy: It is not comfortable waiting in the queue, it makes you anxious because you are uncertain of what will transpire in those rooms, especially when others have finished and you are still waiting your turn and anticipating what is going to happen to you. Waiting gives you pressure and more fear.

Boy: It is very irritating because you are intimidated by the fact that you are not sure of your results and you wait long in the queues, you find out that some even leave without testing because of the queues.

Learners raised concerns over the type of test being administered. The fear of needles was found to be a considerable source of concern for some, while others showed a lack of knowledge about tests that do not require a blood sample.

Girl: I personally am very uncomfortable with the finger prick test. For me it is related to injection of which I am scared. One of the reasons why I do not like the finger prick test is because once I bleed it takes a while for the blood to stop. If there was another way of testing besides the pricking, though I do not know if it exists, but I will be more comfortable with it compared to being pricked, because that is a bit hard for me.

Boy: I also prefer the finger prick test because you can see the drop of your blood being on the testing kit, the results are also obvious before you, meanwhile with the mouth swab, I do not have much idea on how it functions without blood.

Confidentiality is paramount in the views of learners, especially in relation to who performs the test, where the test is conducted, and access to ARVs. A lack of perceived confidentiality stems from a deep suspicion that healthcare workers may violate their privacy and anonymity by informing parents and/or teachers of a leaner’s HIV status, particularly in the event of a positive test.

Girl: Let me say there is this particular lady who works at the clinic who is also a neighbour, this lady might see that particular person going to do an HIV test and tell the family, she now has to explain at home why she or he went for an HIV test.

Girl: I think the people who test us [at school] tell the teachers about our results. Then the teachers run back to us and disclose our results without being informed by the people who tested us. That’s one of the reasons that made us not test.

Boy: One of the community workers disclosed someone’s status while she was under the influence of alcohol. It would be better if we could have community workers who are not living in our area.

The location of the test was a concern to learners who felt that privacy and confidentiality could be undermined. Some learners were concerned that being seen leaving a mobile testing station at school in front of their peers could lead to harmful speculation and discrimination. Moreover, particular behaviour or body language for those tested at school could also lead to unwanted attention.

Boy: The reason why they didn’t test, it was because the testing was done at school. Although pupils would never know if you are HIV positive or negative but they were afraid of students that they may speculate and whenever you get sick they might think you were tested HIV positive.

Girl: …when we are tested here at school, it’s in the classroom. So now say you find out that you are positive, naturally you are in shock, so your schoolmates notice that you are upset when you come out. Then they start speculating that you are positive. So I’d rather we tested elsewhere where there is enough space between the tents. Like at a sports field, where you can come out of one tent and leave if you want to without drawing anyone’s attention.

These concerns lead to a declared preference for different venues, such as hospitals or even at home. It is worth reiterating here that these choices were driven by perceptions about confidentiality, not convenience.

Girl: I also prefer going to hospital and no one will see me when I go there. There are caravans that go around the community. Those clinics just stop wherever they want to, even on the road. So, if you test there everyone will be looking at you. That’s why I desire to go to hospital.

Boy: What will make it easy for some, is that those conducting HIV test should consider doing it at their homes, testing them at their homes, do that one is more comfortable knowing his or her status being at home.

Girl: One other contributing factor on why some are intimidated to visit the local clinic is the system used at the clinic, that all those coming for an HIV test are separated from those who are there to collect their general illness tablets. This is the main reason why one would not go to the clinic.

The prospective thought of dealing with a positive result led to learners revealing additional fears around their anonymity when collecting any required ARVs. This need for confidentiality surrounding the collection of drugs is as important as HCT.

Boy: They are scared to collect the [ARV] medication in the area, because everyone knows that if you are coming from that room. It means you are collecting the medication.

The fear over the consequences of taking ARVs is further expressed through the experiences of those in the community known to be positive and on ARVs. This interaction within the community and the witnessed stigma attached to those on treatment has resulted in learners wanting to avoid the consequences of a positive result.

Girl: I’ve heard people talking about the fear of taking ARV’s. Some of them said ARV’s changes your body shape, some of them said if you take ARV’s you might never able to have children again or they may have mentally disabled kids. People who take ARV’s for the first time, they likely to show signs of a mentally disturbed person, and people are afraid of this.

Girl: Some are scared of treatment because it is said that sometimes when you take treatment, it alters your shape. Like your stomach gets bigger and you don’t look the same. So they are scared that if they are positive and then start taking treatment, they will be easily identifiable because of the change in body shape. And some people don’t speak well of people with HIV so that might also scare people off from testing.

## Discussion

This study is important for two related reasons. It identifies barriers to and facilitators of testing for young people both at the individual and community level, and thereby highlights requirements for the success of strategies to integrate HCT into the wider ISHP. SCT provides a framework for understanding how perceptions about self-efficacy as well as goals and outcome expectancies, which are influenced by personal, interpersonal and environmental factors, as well as by behaviour, will ultimately affect demand for HCT services in schools. The interactions between factors at each one of these levels is particularly important for understanding why learners may or may not be willing to test.

A key issue increasing the fear of a positive result is an individual’s own sexual behaviour. Learners who perceived themselves to be more likely to have contracted HIV because of their risky sexual behaviour were found to be more reluctant to test, highlighting the interaction between beliefs and behaviour, and consistent with existing literature [25, 28, 26]. Learners often said they would rather not undergo testing than risk finding out that they were infected. This fear can be compounded by a lack of knowledge of how to manage a positive result. Participants expressing feelings of fear and hopelessness relating to a positive test result perceived a lack of support from the community in general, as well as an inability of the healthcare system to provide adequate care and support. For some, there were doubts about the availability and effectiveness of HIV medication. Such feelings of fear could be grounded in a lack of knowledge dissemination and education, a bad experience at a healthcare facility, or a damning report from a third party (friend or peer).

Fear of a positive result was also shown to increase because of concerns about taking HIV medication and beliefs about potential side effects [38]. For some, these concerns related to the possible health complications associated with ARVs and decreased quality of life, while for others, the effects of the drugs on body shape and the associated stigma emanating from peers and the community were a concern. This fear of receiving a positive result and having to live with HIV is highly correlated with a lack of knowledge as well as a perceived lack of support from friends, family and the community at large.

The fear of HIV-related stigma and discrimination, well documented in existing literature [25, 28] and supported in this study, acts as strong barrier to testing for individuals. Instead of undergoing HCT as part of a sexual health routine, some individuals would rather not test due to the associated stigma. Previous studies found that learners of self-declared low risk were often unlikely to undergo testing [24, 27] if they felt certain they could not possibly be infected [23]. These learners included those who were not sexually active but also, rather worryingly, those who felt they could trust their intimate partners. While it may be reasonable for these individuals to decline testing, this may also, in part, be driving the social stigma and discrimination around testing because of a lingering perception that only people engaging in unsafe sex need to undergo HCT. Individuals in lower-risk groups may fear being tarnished with the same high-risk brush if they are seen queuing for HCT, and those at high risk may decline testing to avoid being labelled or judged by their family or peers. This is an especially important barrier in Vulindlela, where prevalence rates in school are relatively low, but risk increases dramatically as youth enter their twenties [30, 34].

The threat of HIV-related stigma from friends, family and the community increases the fear of receiving a positive result. This fear of stigma, which results from individuals’ perceptions about the lack of support networks from family, society and the healthcare system, creates a number of other barriers to testing, which may not exist if perceived stigma and discrimination were diminished.

The perceived lack of confidentiality was a particular barrier expressed by participants. This supports previous studies where young people access sexual and reproductive health services in general [32, 39] and HCT specifically [24, 25, 31], although much of this work focuses on the confidentiality of test results. This study emphasises that perceptions about the reasons for confidentiality breaches do vary between learners. Some showed a lack of trust in healthcare workers, while others were more concerned about the privacy of the HCT setting and that their own reactions would compromise the confidentiality of the results. Other learners had concerns about being seen to be testing, highlighting the fear of discrimination, judgement and community stigma surrounding sexual practices. These subtle differences pose potential problems for designing a universal HCT service that will encourage all learners to undergo testing.

Different locations pose various potential threats that relate to perceptions about the confidentiality of HCT, and the test result. A particularly important finding from this study is that the location of HCT is not a proxy for convenience, but rather is related more closely to confidentiality. While it might be expected that increasing the convenience of HCT for learners would increase uptake, participants in this study indicated that confidentiality and proper support were more important considerations. While previous studies found a perception that young people found clinical settings inappropriate for youth [40], this was not noted among participants in this study—a further indication that confidentiality rather than convenience is driving their choice. Scaling up of HCT services in South Africa for the general population is likely to require more dedicated HCT facilities and healthcare workers that deal with large numbers of clients. However, separating HIV services in clinics can lead to increased stigma and negative perceptions about people living with HIV—a real concern for increasing uptake.

Knowledge about HIV and HCT, and the support systems in place, including family, friends and the community at large, were found to be critically important in influencing learners’ decisions to test. In many cases, support from family, peers and friends acts as a strong interpersonal facilitator mitigating against general social stigma and discrimination attached to being HIV positive, as found in previous studies [25, 29]. However, these relationships may also be a significant barrier to HCT if stigmatisation of and judgemental attitudes towards people living with HIV exists, especially from parents. High school learners are at a critical point in their development and it is important not to negate the effects of self-image and peer pressure on their choices [41]. These learners are mostly minors, and the influence of family support, perceptions and values on their own beliefs and behaviour is important. A crucial component in creating a supportive environment, increasing knowledge, and changing beliefs and perceptions about HIV and testing, is counselling, offered by the healthcare service providers. Many learners expressed the importance of counselling in helping decrease the fear of living with HIV and managing a positive status. Understanding the needs of young people and providing appropriate counselling services will be important for the implementation of any successful scale-up programme.

A further barrier to testing found in this study is the fear surrounding the test itself. The aversion to needles found among participants seems to be a common fear among many young people [42, 43]. Offering different types of tests that do not require blood samples for preliminary HIV screening could be a significant facilitator of HCT for young people. For example, OraQuick, approved by the American Food and Drug Association (FDA) is a rapid test requiring only an oral sample [44]. However, an education programme as an antidote to misinformation as to how these tests work, their availability and efficacy, will be necessary if this is to be a successful demand-creation strategy.

All of the barriers reported by the learners in this study linked back to a fear of testing, but specifically, the stigma attached to testing and the fear of a positive result. This fear was found to be the result of a number of factors at the personal, social and environmental levels, with the behaviour of individuals influencing the way they view testing, as well as the way they perceive HIV-related stigma and discrimination from those around them. When learners felt these fears were allayed, either because of their environment or a change in their own views and beliefs about HCT, this was found to increase willingness to undergo HCT.

### Incorporating HCT into the integrated school health policy (ISHP)

Scaling up health service delivery to young people in South Africa has become an increasingly important goal for policy makers. Indeed, strengthening the delivery of healthcare to learners in South African schools is a key component of the NDOH plans to strengthen primary healthcare, as well as being a fundamental part of the Care and Support for the Teaching and Learning programme in the education sector [18]. Furthermore, HCT is one of the targeted interventions for inclusion in the new South African ISHP. However, the implementation of adolescent-targeted programmes and policies can be challenging. Youth are in a critical stage of development, both biologically and cognitively, putting them at increased risk because of poor sexual health outcomes and proving difficult to reach them with existing service delivery mechanisms [41]. The existence of social factors specific to young people can make the provision of sexual health services difficult [45]. Furthermore, the cultural norms vary vastly between different areas in South Africa making the development of standardised interventions for high school learners even more challenging [46].

Integrating HCT into a comprehensive package of health services offered to young people as part of an initiative that reaches out to schools, provides some unique opportunities to reduce barriers to testing. However, it is not without potential problems. The HIV-related stigma and discrimination that exists in schools reinforces barriers to testing, which must be reduced or eliminated in order to facilitate uptake. Offering HCT as part of a range of general healthcare services could help to destigmatise testing in schools by inculcating a culture of HCT, regardless of lifestyle and sexual behaviour, as well as helping to normalise testing. By reducing stigma associated with HIV testing, fears relating to the possibility of a positive result can be reduced, and for learners who perceive themselves to be at high risk, this may reduce many of the barriers to testing discussed in this paper. Reducing stigma and normalising testing will also help encourage learners who do not feel they are at risk to make testing part of their sexual health routine before their risk increases as they transition into adulthood.

Changing stigma is unlikely to happen overnight, and thus must be done in parallel with scaling up service provision. The design of policy and especially the design of the HCT service package offered to learners must take the aforementioned barriers into account.

Comments from participants show that testing at schools presents challenges for preserving confidentiality – especially a problem because of stigma related both to HIV and to HCT. While previous studies have shown that mobile HCT services offered in schools can help to increase uptake [33], poorly managed school testing could act as a barrier to uptake as alluded to by participants. Offering HCT at school means that learners will know who else is testing and are likely to be able to see their reaction after they have finished testing. Including HCT in the ISHP is likely to cause major problems if HIV-related stigma exists, and if learners do not perceive the service to be confidential. A further consideration must be the selection of healthcare workers who provide these services. In this study, learners expressed concern relating to the lack of trust in healthcare workers. Using healthcare workers from within the community may prove to be a significant barrier to the inclusion of HCT in the ISHP.

One of the problems of existing HCT programmes leading to a lack of knowledge of HCT, is that leaners are only counselled once they make the decision to test. By including HCT in the ISHP, pre-test counselling can take place sooner within a general health setting before a decision to test is taken. This increase in knowledge and confidence in the HCT process is likely to act as an individual facilitator. In reality, while counselling is offered to everybody who undergoes HCT, counsellors may have different levels of training, and have different attitudes towards clients [40]. There is a need for more research into what high school learners want and need from the counselling that accompanies testing for HIV.

The effects of peer pressure and the influence of friends and family are well documented [25, 28, 29] and this study adds to that body of literature. Peer educators and peer recruiters have been used as a way of increasing awareness and uptake of HCT. However, for most learners in this study, perceptions about their effectiveness were fairly negative, and learners indicated that teachers had more of an effect on their intentions to test. Including HCT into the ISHP provides an avenue to further increase the influence teachers have on increasing uptake.

While including HCT into the ISHP presents an opportunity to target a captive audience, there are a number of potential threats that could negatively influence testing uptake if services are not carefully designed. Understanding the levers that exist in the interaction between personal-level factors and the social context, as well as the feedback between these factors and individuals’ own behaviour, is vital for the design of services that will be effective in decreasing the fear associated with testing and HIV-related stigma.

#### Limitations

A tension existed between the richness of the data and the need for perceived confidentiality and anonymity of participants. In order to help learners feel more comfortable to discuss what may be perceived to be sensitive topics, information regarding participants’ age, HCT history, HIV status and other important variables were not collected. While this presents a potential limitation of the richness of the data and depth of analysis, assuming homogeneity in the perceptions and preferences of participants in this study seemed reasonable and in line with findings from previous studies highlighting the specific needs of youth [31, 39, 41]. Schools were selected to ensure a representative sample from a community with very similar types of schools servicing learners with similar socio-economic and cultural backgrounds, and learners selected to participate in the study were in a small age range, all in their final 3 years of high school.

Utilising only male fieldworkers could have influenced the interaction and openness amongst female participants. However, after initial discussions were conducted, the fieldworkers and researchers observed that the girls felt comfortable to discuss the questions because of the fairly large discussion groups. The experience of interviewers also helped to mitigate potential problems in the validity of the data, and the study continued using only male fieldworkers, who were trained to be aware of possible sources of bias when conducting the FGDs.

Analysis of the difference in responses between different schools is not reported in this paper as results did not show any such differences. All schools used were situated in rural or semi-rural areas, were under-resourced and located in an area with a high burden of HIV. As a result, the findings in this paper may not be generalisable to the entire population of school-going adolescents. Further, because of ethical issues, learners between the ages of 12 and 16 who are able to consent to HCT were not included in this study.

Participants were not asked to disclose their testing behaviour, their HIV status or any other information that could be perceived to compromise confidentiality. Consequently, learners who had been tested previously were mixed in groups with learners who had not. Participants were encouraged to participate in the discussion based on their perceptions about their peers, experiences of their friends, and if they felt comfortable to do so, their own experiences. Further, at the start of each session, fieldworkers explained the topic for discussion and learners were given the opportunity to opt out at any point in the conversation.

A potential weakness of qualitative research is that the researchers’ own expectations, perceptions and beliefs can influence the analysis of the data. To mitigate this threat to the data, researchers worked as a team to analyse data as objectively as possible. Further, the study was conducted in an area where research is frequently conducted, and respondents may have relatively increased exposure to information and sexual reproductive health services. However, we believe the richness of the qualitative data from learners who have real experiences and understand their preferences makes the findings of this research particularly important, especially in light of the scale-up of education and service provision through the ISHP.

## Conclusions

Fear is the dominant driver of low HCT uptake, and this fear is fuelled by associated HIV-related stigma and discrimination. This presents a significant problem for programme planners and policy makers, because of the number of people needing to be tested annually, and the variety of sources of fear and real or perceived stigma and discrimination. Decreasing stigma by increasing knowledge and fostering an environment in which learners feel they have an adequate support network are key to the success of HCT programmes targeting young people. Increasing education about the importance of HCT, and creating awareness about available HCT services, will not be enough to increase uptake in schools in South Africa. Efforts to decrease stigma around HIV and HCT by integrating testing into general and sexual reproductive health services will go some way to decreasing fears around testing, especially if interventions are sensitive to pervasive personal and social-level factors. Scaling up HCT in schools will require careful planning and strategic marketing and management to increase trust in healthcare professionals, in addition to the integrity and quality of HCT services designed with the specific needs of youth in mind.

## Abbreviations

ART: Antiretroviral therapy
ARV: Antiretroviral
CAPRISA: Centre for the AIDS Programme of Research in South Africa
FDA: Food and Drug Association
FGD: Focus group discussion
HCT: HIV counselling and testing
ISHP: Integrated school health policy
KZN: KwaZulu-Natal
NDOH: National Department of Health
SCT: Social cognitive theory
VMMC: Voluntary medical male circumcision

## Footnotes

1 In this discussion we use this age group as a definition for “youth” in line with much of the literature cited in this paper [39, 41]. Although our research looks only at a sub-population within this group, we discuss the context and literature relating to groups referred to as “youth”, “young people” and “adolescents” and the various ways in which other authors have defined these groups.

2 These additional FGDs were used to augment data for this study and for a concurrent study assessing barriers to and facilitators of VMMC [43]. Since HCT is a prerequisite for VMMC, the FGDs were wholly compatible.
